# Screening left ventricular systolic dysfunction in children using intrinsic frequencies of carotid pressure waveforms measured by a novel smartphone-based device

**DOI:** 10.1088/1361-6579/acba7b

**Published:** 2023-03-01

**Authors:** Andrew L Cheng, Jing Liu, Stephen Bravo, Jennifer C Miller, Niema M Pahlevan

**Affiliations:** 1 Division of Pediatric Cardiology, Children’s Hospital Los Angeles, Los Angeles, CA, United States of America; 2 Division of Cardiovascular Medicine, Keck School of Medicine, University of Southern California, Los Angeles, CA, United States of America; 3 Department of Aerospace and Mechanical Engineering, University of Southern California, Los Angeles, CA, United States of America

**Keywords:** left ventricle ejection fraction, pediatric heart failure, machine learning, cardiovascular intrinsic frequencies

## Abstract

**Objective.:**

Children with heart failure have higher rates of emergency department utilization, health care expenditure, and hospitalization. Therefore, a need exists for a simple, non-invasive, and inexpensive method of screening for left ventricular (LV) dysfunction. We recently demonstrated the practicality and reliability of a wireless smartphone-based handheld device in capturing carotid pressure waveforms and deriving cardiovascular intrinsic frequencies (IFs) in children with normal LV function. Our goal in this study was to demonstrate that an IF-based machine learning method (IF-ML) applied to noninvasive carotid pressure waveforms can distinguish between normal and abnormal LV ejection fraction (LVEF) in pediatric patients.

**Approach.:**

Fifty patients ages 0 to 21 years underwent LVEF measurement by echocardiogram or cardiac magnetic resonance imaging. On the same day, patients had carotid waveforms recorded using Vivio. The exclusion criterion was known vascular disease that would interfere with obtaining a carotid artery pulse. We adopted a hybrid IF-Machine Learning (IF-ML) method by applying physiologically relevant IF parameters as inputs to Decision Tree classifiers. The threshold for low LVEF was chosen as <50%.

**Main results.:**

The proposed IF-ML method was able to detect an abnormal LVEF with an accuracy of 92% (sensitivity = 100%, specificity = 89%, area under the curve (AUC) = 0.95). Consistent with previous clinical studies, the IF parameter *ω*_1_ was elevated among patients with reduced LVEF.

**Significance.:**

A hybrid IF-ML method applied on a carotid waveform recorded by a hand-held smartphone-based device can differentiate between normal and abnormal LV systolic function in children with normal cardiac anatomy.

## Introduction

Ventricular function is one of the fundamental components of cardiovascular health in children with congenital and acquired heart disease. It follows that the ability to detect ventricular dysfunction is critical for clinicians in guiding the treatment, management, and prognosis of children with cardiovascular disease. The transthoracic echocardiogram is the primary imaging modality for assessing the anatomy and function of the heart (Cheitlin *et al* 2003, Lai *et al* 2006). However, echocardiography requires specialized training to perform and interpret, limiting the settings where an echocardiogram can be obtained. While physicians and parents of children with heart disease are taught to recognize symptoms of heart failure (HF), many of these symptoms, such as tachypnea, are nonspecific (Price 2019). Children with HF are also known to have higher rates of emergency department utilization, health care costs, and rates of admission (Shamszad *et al* 2013, Price *et al* 2016, Mejia *et al* 2018). In resource-limited health care settings, such as rural communities, and in the home, an urgent need exists for a simple, non-invasive, and inexpensive method of screening for left ventricular (LV) dysfunction to determine if a patient needs further medical evaluation.

A newly developed wireless smartphone-based handheld device, the Vivio, uses the intrinsic frequency (IF) method to quickly and non-invasively measure LV function (Pahlevan *et al* 2014, Pahlevan *et al* 2017b, Armenian *et al* 2018, Rinderknecht *et al* 2020). The Vivio is an optical tonometer and phonogram that can quickly capture arterial waveforms (Rinderknecht *et al* 2020). This device is non-invasive, inexpensive, easy to use, ultra portable, and compatible with Bluetooth-capable smartphones and tablets (Rinderknecht *et al* 2020). We recently demonstrated the practicality and reliability of the Vivio device in capturing carotid arterial waveforms and deriving IFs in children with normal cardiovascular function (Miller *et al* 2020).

The IF method is a new systems-based mathematical method that considers the arterial network as a dynamic system coupled to the LV during systole and uncoupled during diastole. Intrinsic frequencies (IFs) are operating frequencies that are physically and mathematically different than resonance-type frequencies, such as Fourier frequencies (Pahlevan *et al* 2014, Tavallali *et al* 2015, Alavi *et al* 2021). The IF method can extract information about LV function, vascular dynamics, and the interaction between the LV and arterial system (LV-arterial coupling) from pressure waveforms (Pahlevan *et al* 2014, Pahlevan *et al* 2017b, Tavallali *et al* 2018, Mogadam *et al* 2020). Previous clinical studies have shown that the IF method can be applied to carotid artery waveforms measured by Vivio or a smartphone (an iPhone) to compute LV ejection fraction (LVEF), the most common measure of global LV systolic function (Pahlevan *et al* 2017b, Armenian *et al* 2018). In a recent clinical study based on the Framingham Heart Study (FHS) data, it was shown that IFs derived from a custom tonometer (functionally similar to the Vivio) could be used to predict HF events in adults (Cooper *et al* 2021). Detecting decreased LVEF in pediatric patients early via IF screening would similarly be clinically useful, as ventricular dysfunction has been associated with morbidity and mortality in children admitted with a variety of conditions including sepsis and cardiomyopathy (McMahon *et al* 2004, Fisher *et al* 2005).

In this study, we explored the use of a non-invasive, inexpensive, easy to use, ultra-portable device (Vivio) in pediatric patients with normal cardiac anatomy and depressed LV systolic function, as assessed by transthoracic echocardiogram and cardiac magnetic resonance imaging (MRI). Our goal was to demonstrate that a novel IF-based machine learning (ML) method (focusing on IFs that are linked to LV systolic function such as *ω*_1_ and *φ*_1_) applied on carotid waveforms measured by Vivio can distinguish between normal and reduced (abnormal) LVEF in pediatric patients.

## Methods

### Study design

The study was conducted at Children’s Hospital Los Angeles (CHLA). Patients ages 0 to 21 years who had undergone an echocardiogram or cardiac MRI for clinical purposes were invited to participate in the study. The only exclusion criterion was known vascular disease that would interfere with obtaining a carotid artery pulse. Informed consent was obtained from participants, or their legal guardians for those who were minors. Patients underwent LVEF measurement by either echocardiogram or cardiac MRI. On the same day, patients had a carotid artery waveform recorded using the Vivio device. The study was approved by the CHLA Institutional Review Board (CHLA-17–00377).

### Lvef measurement

Echocardiograms were performed at CHLA using either an IE33 or Epiq 7 ultrasound system (Philips, Best, Netherlands). Studies were performed according to American Society of Echocardiogram guidelines (Lai *et al* 2006, Lopez *et al* 2010). LV systolic function was evaluated by LVEF. IntelliSpace Cardiovascular Workstation (Philips) was used to calculate end-systolic and end-diastolic volumes using the modified Simpson’s method in apical 4 and apical 2 chamber views. LVEF was then calculated as: LVEF = 100*(LV end diastolic volume—LV end systolic volume)/(LV end diastolic volume) (Lopez *et al* 2010).

Cardiac MRI studies were performed at CHLA using a 1·5 Tesla Achieva system (Philips, Best, Netherlands). Images were obtained using a balanced steady state free procession sequence without use of a contrast agent. Each dataset consisted of 15 short-axis slices covering the left ventricle from base to apex with 30 frames per cardiac cycle. Typical scan parameters were slice thickness 6–10 mm, in-plane spatial resolution 1·5–2 mm^2^, repetition time 3–4 ms, echo time 1·5–2 ms, and flip angle 60 degrees. Images were obtained with the patients free breathing; 3 signal averages were obtained to compensate for respiratory motion. Manual image segmentation was performed using Circle cvi42 v.5.10 software (Circle Cardiovascular Imaging Inc., Calgary, Canada). Endocardial contours were drawn on end-diastolic and end-systolic images. LVEF was then calculated from these contours as above.

### Noninvasive carotid artery waveform measurement using Vivio

**Figure 1.**
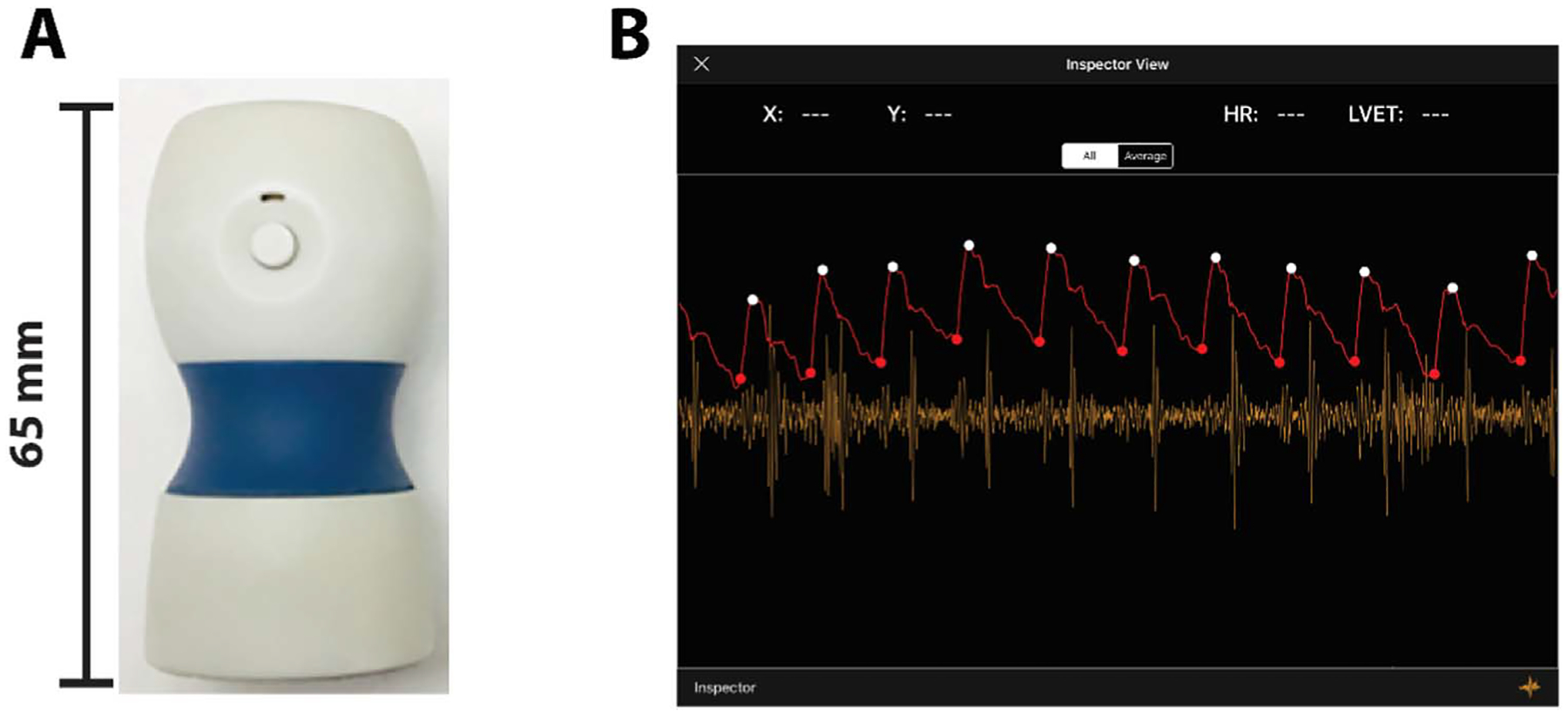
The wireless Vivio system for cardiovascular monitoring. (A) The Smartphone-based device (Vivio). (B) The user interface for real-time patient monitoring of the mobile application for the Vivio.

A trained physician positioned each patient’s head to expose the carotid triangle by rotating their head laterally 30–60 degrees and up 30 degrees. After palpating the common carotid artery pulse, the physician then used the Vivio device to record the carotid pulse waveform. Waveforms were recorded for one minute to ensure that high-quality tracings were obtained over multiple cardiac cycles. All patients were studied at rest. After the waveforms were collected, cardiac cycles were selected by a researcher blinded to study participant clinical history and LVEF data. For each patient, three to five cardiac cycles deemed good signal quality were selected from the Vivio carotid waveforms. The selected cycles were used to calculate the IF parameters (see the next section) and the IF parameters from the selected cycle were averaged to serve as the final IF analysis result for the individual (each patient has only one set of IF parameters). A picture of the Vivio device and sample waveforms measured by it are provided in figure 1.

### Intrinsic frequency method

**Figure 2.**
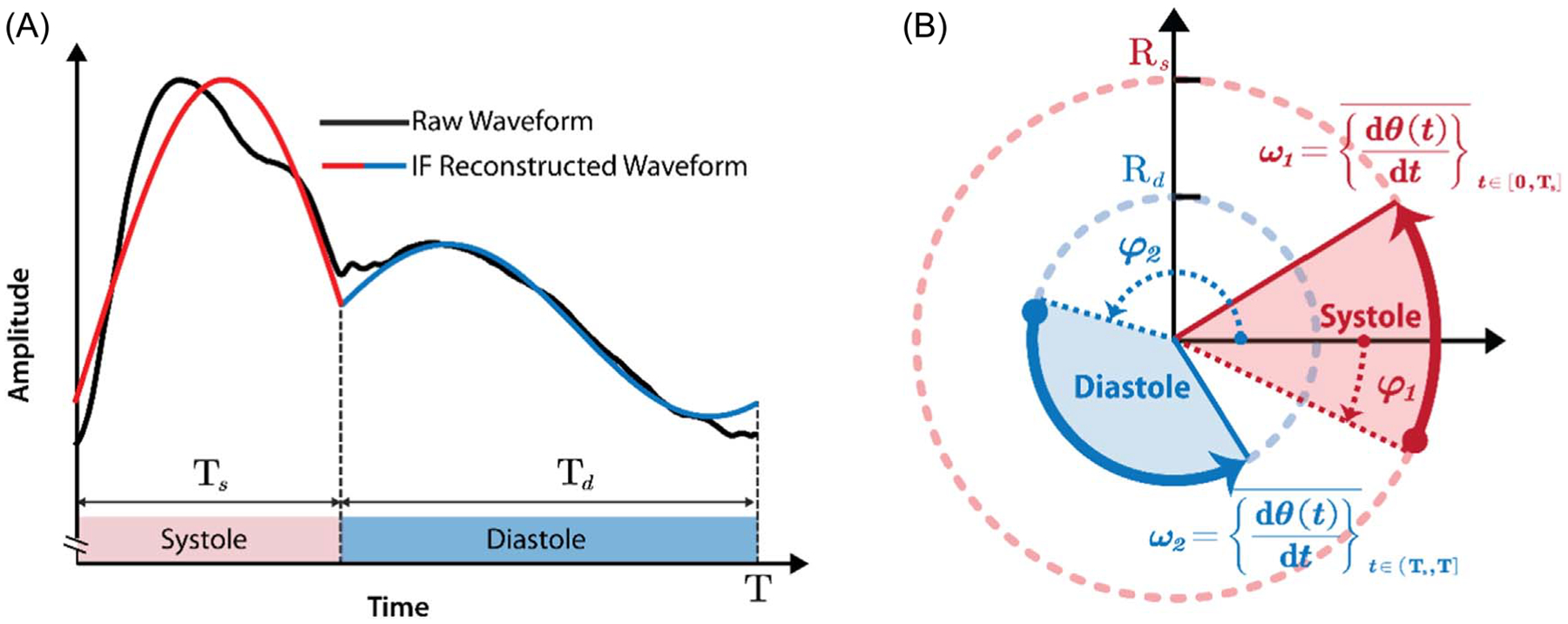
Illustration of intrinsic frequency (IF) method. (A) Reconstruction of arterial pulse with IF method. (B) Visualization of IFs. *ω*_1_ and ω_2_ are the IFs during systole and diastole, respectively. *R*_*s*_ and *R*_*d*_ are the envelopes of IF components associated with *ω*_1_ and *ω*_2_ respectively. *φ*_1_ and *φ*_2_ are the phase shifts (or intrinsic phases) of the IF components associated with *ω*_1_ and *ω*_2_ respectively. T_*s*_ is the systolic time, and it is equal to T_0_. *T*_d_ is the diastolic time, and it is equal to *T*–T_0_.

Intrinsic frequencies are operating frequencies based on the Sparse Time-Frequency Representation (STFR) (Hou and Shi 2011), treating the LV combined with the aorta and the remaining peripheral arteries as a coupled dynamical system (heart + aortic tree), which decouples upon closure of the aortic valve (Pahlevan *et al* 2014, Tavallali *et al* 2015, Pahlevan *et al* 2017b). The IF method models a dynamical system as an object rotating around an origin. The angular velocity of the rotation is the intrinsic frequency (see figure 2). In the LV-arterial system, the average angular velocity during systole and diastole are *ω*_1_ and *ω*_2_, respectively. The first IF, *ω*_1_, describes the dynamics of the systolic phase of the cardiac cycle, where the LV and aorta are a coupled dynamical system (Pahlevan *et al* 2014, Tavallali *et al* 2015, Pahlevan *et al* 2017b). The second IF, *ω*_2_, is dominated by the dynamics of the vasculature (Petrasek *et al* 2015, Pahlevan *et al* 2017b, Tavallali *et al* 2018). Further details about the physics and mathematics of IF can be found in previous publications (Pahlevan *et al* 2014, Tavallali *et al* 2015, Alavi *et al* 2021). The IF mathematical formulation is:

$$\text{Minimize}:\|\text{p}(\text{t})-\chi \left(0,{\text{T}}_{0}\right)[\left({\text{a}}_{1}\text{cos}\left(\omega_{1}\text{t}\right)+{\text{b}}_{1}\text{sin}\left(\omega_{1}\text{t}\right)\right]-\chi \left({\text{T}}_{0},\text{T}\right)[\left({\text{a}}_{2}\text{cos}\left(\omega_{2}\text{t}\right)+{\text{b}}_{2}\text{sin}\left(\omega_{2}\text{t}\right)\right]-{\text{c}}_{2}^{2}\|.$$

Here, p(t) is the carotid arterial waveform and *χ* (a, b) is the indicator function (*χ* (*α*, *β*) = 1 if *α* ⩽ *t* ⩽ *β* and *χ*(*α*, *β*) = 0 otherwise). The initial phases (*φ*_1_ and *φ*_2_) and envelopes (*R*_*s*_ and *R*_*d*_) can be computed from a_1_, *b*_1_, *a*_2_, *b*_2_: *φ*_1_ = tan^−1^(*a*_1_/b_1_), *φ*_2_ = tan^−1^(*a*_2_/*b*_2_), $R_{s}=\sqrt{a_{1}^{2}+b_{1}^{2}}$, $R_{d}=\sqrt{a_{2}^{2}+b_{2}^{2}}$. Here, tan^−1^ is the tangent inverse function. *φ*_1_ and *φ*_2_ are the initial phase shifts (or intrinsic phases) associated with *ω*_1_ and *ω*_2_ respectively. *R*_*s*_ and *R*_*d*_ are the envelopes of IFs related to *ω*_1_ and *ω*_2_ respectively. Reconstruction of an arterial pulse with IF method and visualization of IFs during systolic and diastolic phases are provided in figure 2.

### Parameter selection and analysis

Previous clinical studies have indicated that IF systolic parameters (e.g. *ω*_1_) extracted from carotid waveforms can reflect LV systolic function (Pahlevan *et al* 2017b, Mogadam *et al* 2020). Hence, *ω*_1_ and *φ*_1_, which delineate LV systolic function, were considered as physiologically relevant metrics for classifying low LVEF. In this study, *ω*_1_ was also corrected for LV ejection time. Since heart rates (HR) normally change with age in children (Finley and Nugent 1995), we also considered the *ω*_1_ index, denoted as *ωi*_1_, which is *ω*_1_ normalized with respect to the HR. We expected that *ωi*_1_ would provide supplementary information to *ω*_1_ for predicting low LVEF in our pediatric cohort across all ages.

We previously reported that there was a significant difference in *ω*_1_ among different age groups (Miller et al 2020); particularly, *ω*_1_ of the age group from 0 to 4 years old showed significant difference from the that of the adult cohort (Miller *et al* 2020). Therefore, we divided the study population into three age groups: 0–6 years old, 7–13 years old, and 14–20 years old. Then, we compared the *ω*_1_ and *ωi*_1_ between patients with LVEF <50% and LVEF ⩾50% in each age group. To grasp the classification ability of *ω*_1_ and *ωi*_1_ intuitively, we also used the Beeswarm plots to inspect the scattering and distribution of *ω*_1_ and *ωi*_1_ for the low LVEF and normal LVEF patients in age-separated groups.

### Hybrid IF-machine learning algorithm

In our analysis we adopted a hybrid IF-Machine Learning (IF-ML) method by applying physiologically relevant IF parameters (i.e. *ω*_1_, *φ*_1_, and *ωi*_1_) as inputs to Decision Tree classifiers. Decision Tree is a well-established machine learning approach that exhibits good interpretability through generating a set of if-then-else decision rules which can be visualized as a binary tree (Lewis 2000, Song and Ying 2015). The decision tree technique can facilitate the exploration and development of classification criteria for clinical decision making based on relevant IF-derived parameters and their physiological connotation.

We used the IF-ML method to identify low LVEF in our pediatric cohort (*N* = 50) based on the physiologically relevant feature space composed of *ω*_1_, *φ*_1_, *ωi*_1_, and age. The threshold for low LVEF was chosen as <50%, and positive labels were assigned for patients with low LVEF. The Statistics and Machine Learning Toolbox of MATLAB (The MathWorks, Inc., Natick, Massachusetts) was used to create the binary decision trees using the standard CART algorithm (Lewis 2000). To illustrate the exemplary decision rules based on the novel IF parameters for classifying low LVEF, we presented the tree structures developed based on the whole dataset set. To strive for concision of the decision tree and mitigate overfitting, we set the constraints that the maximal number of the tree splits cannot be greater than (1 + number of predictors), and that the samples in the splitting nodes should be greater than 10.

### Validation and analysis

We employed leave-one-out cross-validation (LOCCV) in our dataset to evaluate the classification performance. Considering the sample size, we chose the leave one out cross-validation (LOOCV) for fair comparisons between different feature combinations. LOOCV does not overestimate test error rates and it gives the same estimate because the partitions are not chosen randomly. The analysis metrics include the area under curve (AUC) in receiver-operating characteristic (ROC) analysis, sensitivity, specificity, and accuracy, which are defined as:

$$\text{Sensitivity}=\frac{\text{True Positive}}{\text{True Positive}+\text{False Negative}},$$

$$\text{Specificity}=\frac{\text{True Negative}}{\text{True Negative}+\text{False Positive}},$$

$$\text{Accuracy}=\frac{\text{True Positive}+\text{True Negative}}{\text{All Positive}+\text{All Negative}}.$$

## Results

### Patient cohort

**Figure 3.**
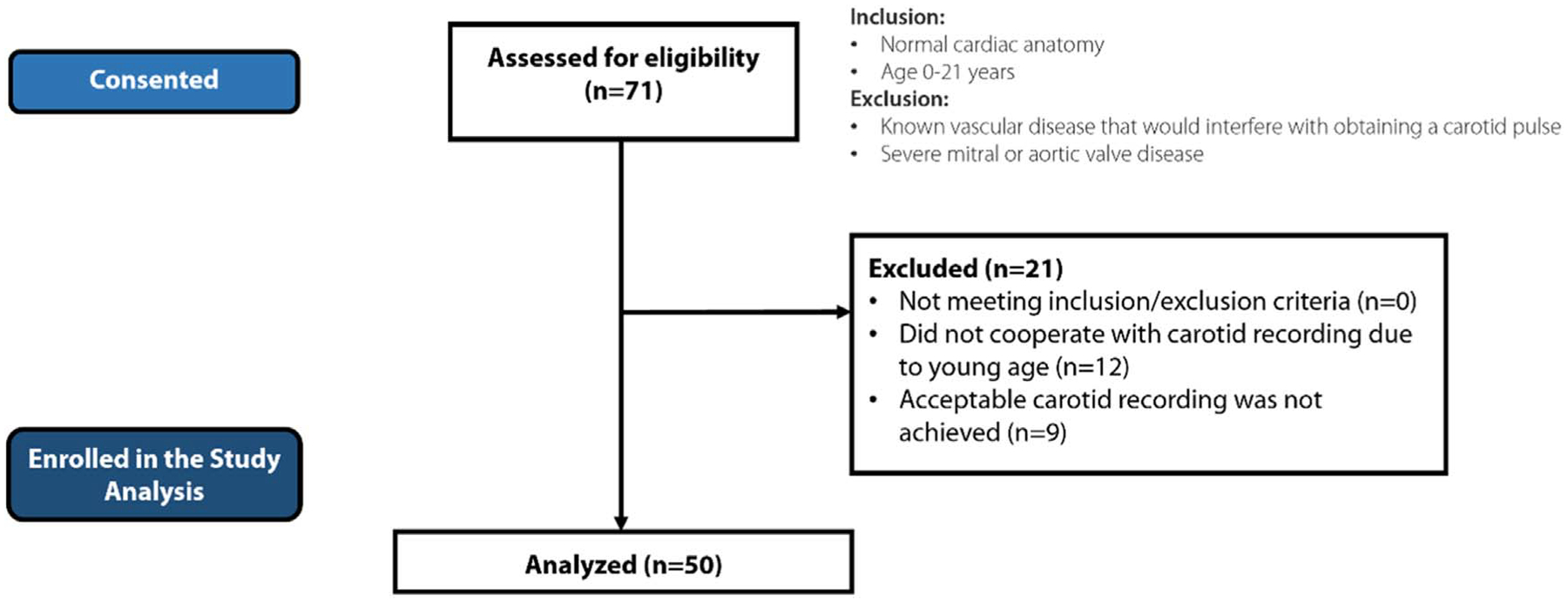
The flowchart of patient enrollment process.

Figure 3 summarizes patient enrollment. A total of 71 pediatric patients’ guardians were consented (1/3/18 to 11/6/19) for this study. Due to their young age, twelve patients did not cooperate with recording of their carotid pressure waveforms with the Vivio device. Acceptable carotid tracing measurements were not achieved in nine patients (low signal quality and severe distortion of carotid waveforms due to body motion or respiratory motion). For the remaining consented patients, signal quality was confirmed manually by one of the study investigators (N.M.P.) before any analysis and calculations of intrinsic frequencies. Thus, 50 patients were included in the study analysis. Table 1 summarizes the demographic characteristics of the patient cohort. No patients had significant aortic valve disease, aortopathy, or hypertension. Forty-six had LVEF measured by echocardiogram and 4 had LVEF measured by cardiac MRI. Of the patients evaluated by echocardiogram, 32/46 (70%) had normal LVEF and 14/46 (30%) had abnormal LVEF. Of those evaluated by cardiac MRI, 2/4 (50%) had normal LVEF and 2/4 (50%) had abnormal LVEF.

**Figure 4.**
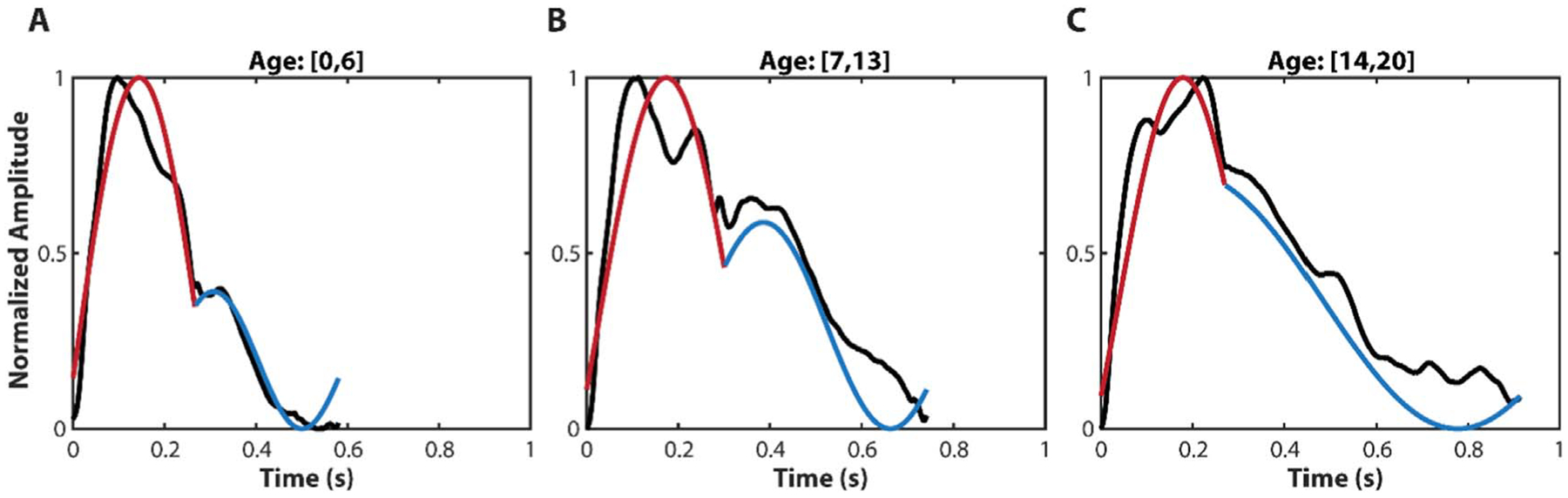
Representative raw carotid waveforms (black curve) measured by Vivio, and IF reconstructed waveforms (red curve: systolic phase; blue curve: diastolic phase) in different ages. (A) sample IF reconstruction waveform for a 5 year old patient. (B) sample IF reconstruction waveform for a 9 year old patient. (C) sample IF reconstruction waveform for a 19 year old patient.

**Figure 5.**
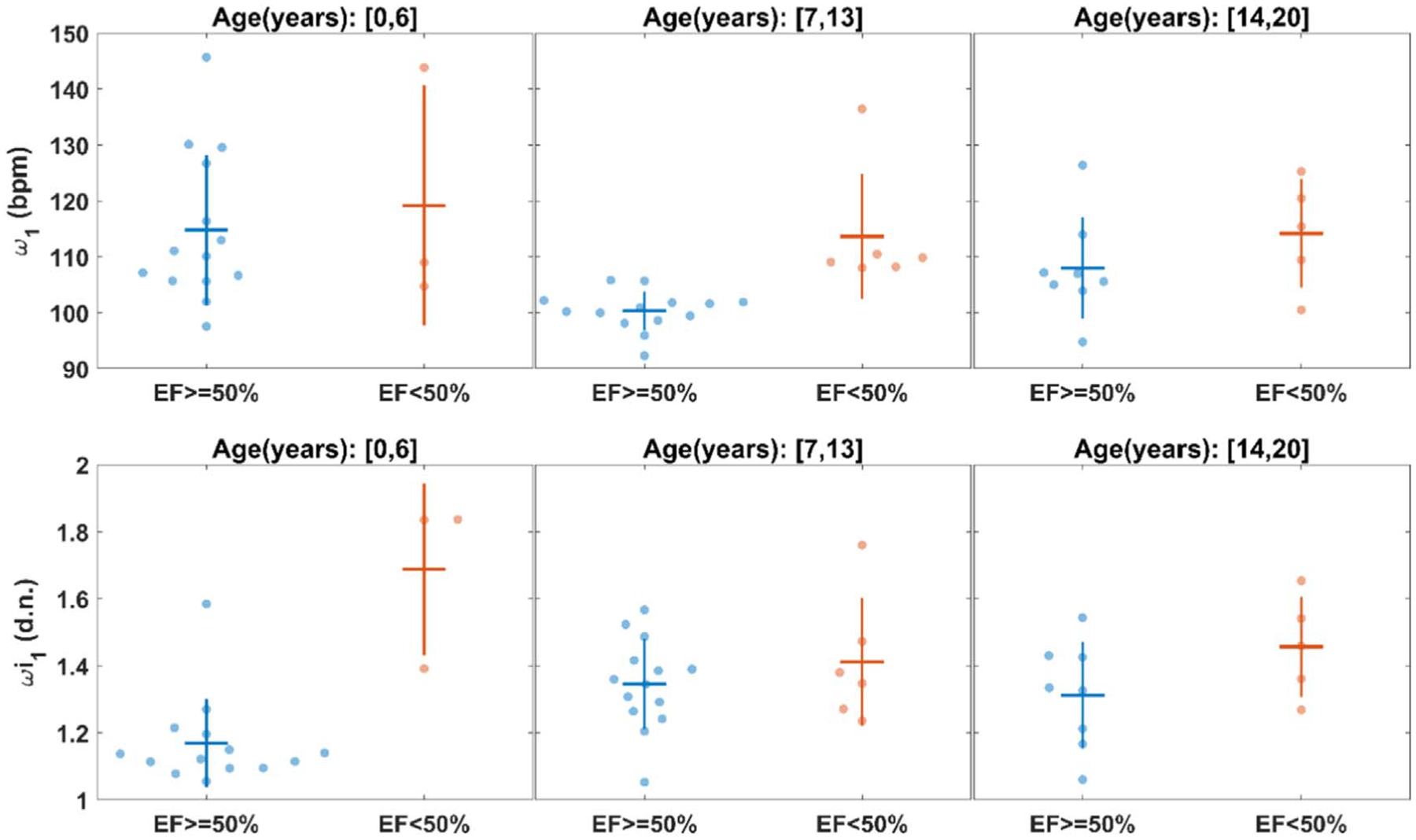
Beeswarm plots for the scattering of *ω*_1_ and *ωi*_1_ in different age groups and LVEF (EF). The cross overlying the scattering dots indicates the mean value ± standard deviations for the group of samples. Unit of *ω*_1_ is beats per minutes (bpm). *ωi*_1_ is unitless.

Reconstruction of sample waveforms from IF analysis (the systolic phase is the red curve and the diastolic phase is the blue curve) with the corresponding raw pulse waveforms (black curve) are shown in figure 4 for patients in different age brackets. The Beeswarm plots in figure 5 show the distribution of *ω*_1_ and *ωi*_1_ in different age groups and EF levels. The supplementary predictive power of *ωi*_1_ to *ω*_1_ can be perceived from figure 5. We can observe that there is a larger discrepancy of *ωi*_1_ in the age group of [0, 6] (years) between the normal and abnormal low EF groups.

### Reduced LVEF detection based on *ω*_1_, Age, and *φ*_1_

**Figure 6.**
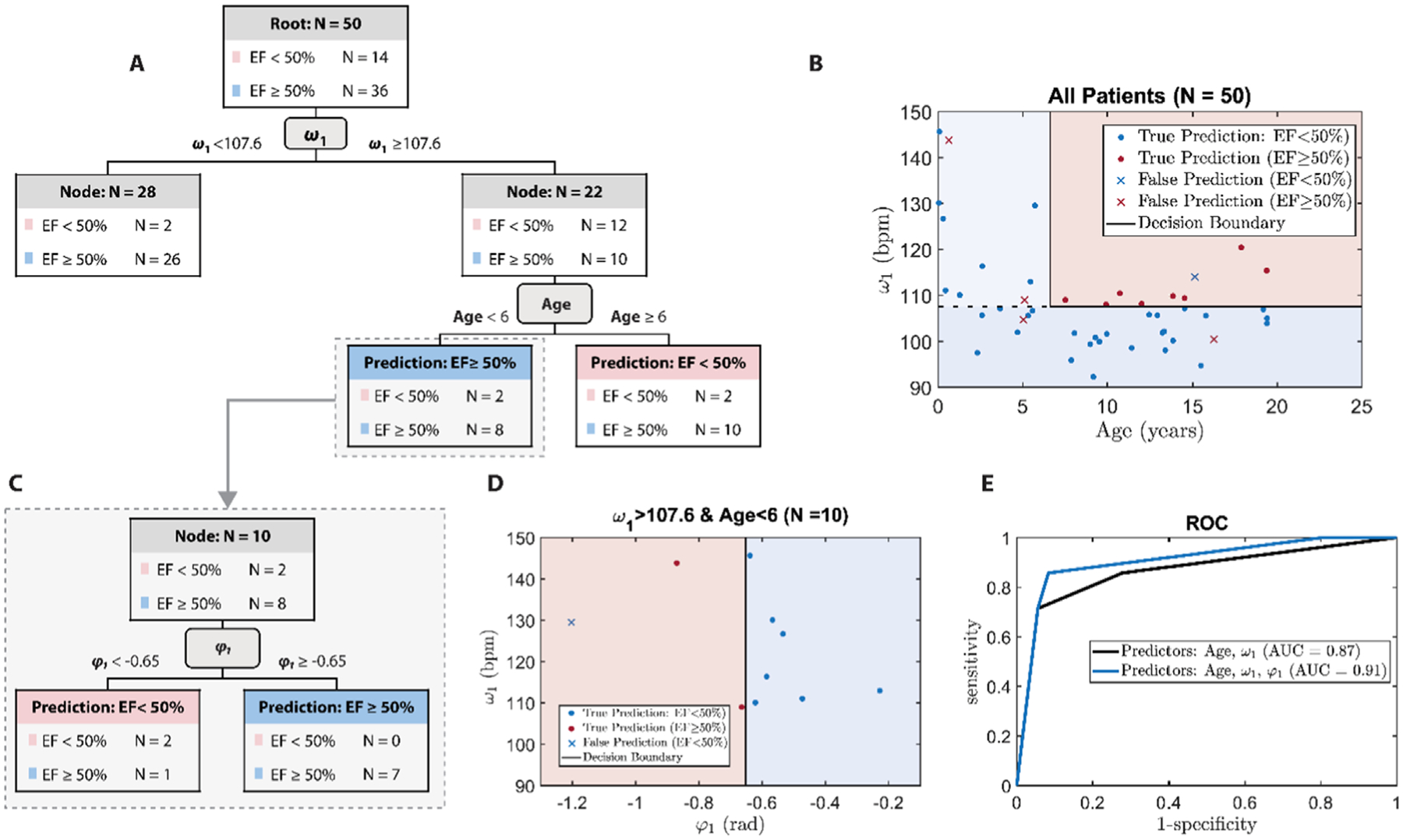
Decision tree analysis for low LVEF (EF) classification (<50%) with IF parameters and age. (A) The decision tree for classifying low EF (<50%) using *ω*_1_ and age. (B) Visualization of the decision boundary and classification result based on *ω*_1_ and age. (C) The expanded branch from the node of patients with *ω*_1_⩾107·6 and age <6 (*N* = 10) for leveraging *φ*_1_ for further classification of low EF patients. (D) Visualization of the decision boundary based on *φ*_1_ for classifying low EF patients with *ω*_1_⩾107·6 and age <6. (E) The ROC of decision tree classifier based on the predicting features of *ω*_1_ and age (black line), and the predicting features of *ω*_1_, *φ*_1_, and age (blue line). The units for *ω*_1_ is bpm (beats per minute). *φ*_1_ is in radian.

Figure 6(A) shows the decision tree for classifying low LVEF (<50%) patients with age and *ω*_1_ developed for the whole study population (*N* = 50). The splitting boundary for *ω*_1_ and age are 107·6 bpm and 6 years old (extracted by the IF-ML algorithm), respectively. The decision boundary based on *ω*_1_ and age is depicted as the black line in figure 6(B). It can be seen in this figure that no decision rule was grown for the low LVEF for patients younger than 6 years old. By integrating *φ*_1_ into the tree structure, an expanded branch with *φ*_1_ as the splitting node grew for patients (*N* = 10) with *ω*_1_> 107·6 and age <6. Figure 6(C) visualizes the branch with *φ*_1_ as the splitting node with *φ*_1_ = −0·65 as the decision boundary. The ROC curves for the decision trees based on the predictor sets [*ω*_1_,age] and [*ω*_1_,age, *φ*_1_] are demonstrated in figure 6(E).

### Reduced LVEF detection based on *ω*_1_ and *ωi*_1_

**Figure 7.**
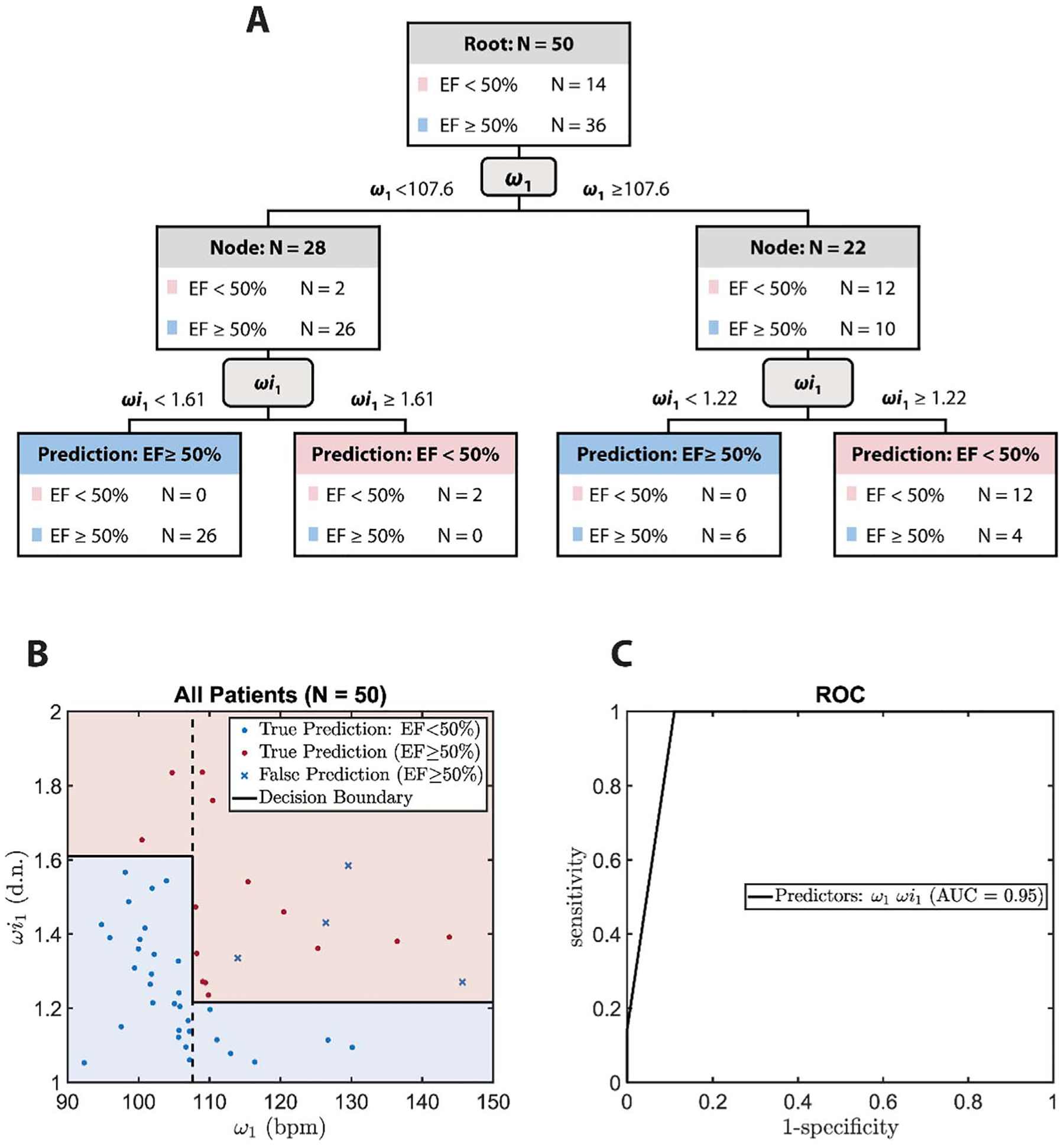
Decision tree analysis for low LVEF (EF) classification (<50%) with IF parameters only. (A) The decision tree for classifying low LVEF (<50%) using *ω*_1_ and *ωi*_1_. (B) Visualization of the decision boundary and classification result based on *ω*_1_ and *ωi*_1_. (C) The ROC of decision tree classifier based on the predicting features of *ω*_1_ and *ωi*_1_. The units for *ω*_1_ is bpm. *ωi*_1_ is unitless.

Figure 7(A) shows the decision tree for LVEF classification using *ω*_1_ and *ωi*_1_. The results indicate that *ω*_1_ = 107·6 bpm still serves as the top-level splitting boundary. *ωi*_1_ further subdivided each branch as shown in figure 7(A). According to the decision boundary visualized in figure 7(B), the upper right region highlighted in red is classified as low LVEF, which is associated with high *ω*_1_ and *ωi*_1_, as compared to the blue region, classified as normal LVEF. The ROC curve for the decision tree with *ω*_1_ and *ωi*_1_ as the predictors is depicted in figure 7(C). The AUC was 0·95, as shown in figure 7(C).

### Classification performance of IF-based models

Table 2 summarizes the classification performance of the decision trees when different systolic IF features are selected. Sensitivity, specificity, accuracy, and AUC for IF-ML analyses are provided in this table. The leave-one-out validation analyses of the IF-ML models are also presented in this table. While the addition of *φ*_1_ can further improve the performance as compared to the feature set of [*ω*_1_, *age*] in full sample analysis, it does not hold in the leave-one-out validation analyses. The feature set of [*ω*_1_, *ωi*_1_] produces the best classification performance in terms of AUC in both full sample and leave-one-out validation analyses. This is further discussed in the Discussion section.

## Discussion

In this study we demonstrated that IFs derived from a carotid artery waveform captured by the Vivio device can be used to discriminate between normal and abnormal LVEF in children with normal cardiac anatomy. Overall, we observed that in all age groups, higher *ω*_1_ and *ωi*_1_ were associated with low LVEF on echocardiogram or cardiac MRI. We then tested several decision tree models that combined the different IFs and clinical variables to develop a hybrid machine learning (ML) model to predict if a patient had normal or abnormal LVEF. Our hybrid IF-ML method was able to detect an abnormal LVEF with an accuracy of up to 92% (sensitivity = 89%, specificity = 100%, AUC = 0·95). Consistent with previous clinical studies (Pahlevan *et al* 2014, Pahlevan *et al* 2017b, Mogadam *et al* 2020), *ω*_1_ was elevated among patients with reduced LVEF (figure 5); hence, it played a dominant role in IF-ML models for low LVEF detection as expected.

The interpretation and usage of our IF-ML models are straightforward. When age and *φ*_1_ were used along with *ω*_1_, *ω*_1_ <107·6 was indicative of normal LVEF regardless of age and the value of *φ*_1_. However, *ω*_1_ >107·6 only confirmed abnormal LVEF in patients older than 6 years. For children younger than 6 years, abnormal LVEF was confirmed via reduced *φ*_1_. For this age bracket (younger than 6 years), only those with high *ω*_1_ (>107) and low *φ*_1_ (< −0·65) had abnormal LVEF. It is noteworthy, that the age of 6 years as a branching point was not pre-enforced, but was deduced by the algorithm itself. Although using only *ω*_1_ and age created an accurate (88%) classification of LVEF with AUC of 0·87, as shown in table 2, both accuracy and AUC were improved when *φ*_1_ was added as a classifying parameter. However, the accuracy was slightly reduced under leave-one-out validation analysis when *φ*_1_ was included. This may suggest that the augmented classification ability by *φ*_1_ could be unstable to dataset/training population and it will require a larger dataset for further investigation.

As demonstrated in figure 7 and table 2, the IF-ML algorithm was able to create a more accurate, more specific, and more sensitive model for LVEF classification when *ω*_1_ and *ωi*_1_ were considered. The model’s robustness is evident by its simplicity, symmetry, and high AUC under leave-one-analysis validation test. Based on this model, *ωi*_1_ >1·6 and *ωi*_1_ < 1·22 indicate abnormal LVEF and normal LVEF, respectively. For values of *ωi*_1_ between 1·6 and 1·22, classification of LVEF is decided by the value of *ω*_1_, where *ω*_1_ >107·6 corresponds to low LVEF (note that this *ω*_1_ threshold is the same as the previous model where age and *φ*_1_ were used). The IF-ML model based on *ω*_1_ and *ωi*_1_ was universal for all pediatric ages. This was achieved by incorporation of HR (a strong correlate of age among pediatric population) through *ωi*_1_.

Given the hemodynamic, physiologic, and anatomical differences between infants, toddlers, school age children, and teenagers, making standardized or predictive models can be difficult for pediatric patients. It is common, if not expected, for other diagnostic studies such as echocardiogram, plain film radiography, and blood tests to need adjustment for age to interpret results (Gutgesell *et al* 1977, Dallaire *et al* 2016, Cantinotti *et al* 2020). While not a comprehensive diagnostic modality such as an echocardiogram or cardiac MRI, the Vivio device and IF method can potentially serve as a powerful adjunctive tool in a variety of clinical scenarios. In resource limited settings, rural clinics, or at home, if a child with heart disease becomes sick, the decision to wait and see if a child improves or seek advanced cardiac care may be influenced if abnormal LV function is detected. The explosion of telehealth services in the past year has also created the need for new technology (Burke and Hall 2015, Sasangohar *et al* 2018). Several patients and family members in our study expressed enthusiasm and interest in having the device at home. Home use of the Vivio device based on the proposed ML-based IF model could allow cardiologists and families to remotely monitor a child’s cardiac function, providing more valuable care via telehealth.

Similar to other tonometry devices, the Vivio is a virtually risk-free diagnostic modality. Empirically, we observed that the Vivio device was minimally intimidating and well tolerated in this pediatric population. In addition, capturing the carotid waveform is a quick and painless process. While we collected 1 min of arterial waveforms during our study and computed IFs for multiple cardiac cycles, only one cardiac cycle is needed to instantaneously estimate LVEF qualitatively. Comparatively, an echocardiogram would take a technician on average 30 min to perform bedside, not including the time needed to physically transport the equipment or patient to and from the scan, and additional time for the study to be interpreted by a cardiologist (Lai *et al* 2006).^2^ The ease of use of the device also allows parents and non-cardiac medical providers to be taught how to use the device. Our proposed ML-derived IF method combined with the Vivio device may also become a cost-effective screening tool in patients who need serial assessments of LV function, such as children who have undergone heart transplantation. Critical care is another area where the Vivio and IF measurement estimations of LVEF would be very useful. Similar to the utility of continuous arterial blood pressure monitoring, continuous monitoring of LVEF, derived from IF calculations from these same arterial blood pressure tracings, could be of great utility in detecting early improvement or worsening of cardiac function. This information would help guide providers whether other studies such as echocardiogram are indicated, if cardiac support should be increased, or if cardiac support could safely be weaned.

### Limitations

A limitation to this study is the relatively low number of patients enrolled in each age bracket. Since each age bracket has its own normal heart rate, they will have different dynamical states. Consequently, each age bracket has its own IF equation that estimates LVEF quantitively. Future studies will be aimed at deriving IF equations that can estimate LVEF (as opposed to only classifying it as normal versus abnormal) similar to what has been done with the Vivio device or the iPhone camera in adults (Pahlevan *et al* 2017a, 2017b, Armenian *et al* 2018). Howeverthis will require enrolling a larger number of patients in each age bracket. In future studies, we will also examine a larger cohort of pediatric patients with LV dysfunction, in addition to different cardiac anatomies, to further evaluate additional biometrics such as body mass index, clinical variables, and factors such as respiration and motion artifacts that may influence the predictive value of IFs. We will also investigate the classification performance with blinded testing.

While there is known discrepancy between LVEF measurements by echocardiogram compared to cardiac MRI, the majority of the patients in this study had LVEF measurement by echocardiogram. Of the small number who had LVEF measured by cardiac MRI, an even number had normal versus abnormal LVEF. Thus, the difference in LVEF calculation between the two modalities is unlikely to have significantly affected our decision tree analysis of normal versus abnormal LVEF. Also, statistical significance tests were not performed due to limited sample size in some age subgroups and EF categories.

## Conclusion

Our study demonstrated that intrinsic frequencies calculated from a carotid artery waveform recorded by a hand-held smartphone-based device (the Vivio) can be used to differentiate between normal and abnormal LV systolic function (as measured by LVEF) in children with normal cardiac anatomy. Overall, the IF-ML model based on *ω*_1_ and *ωi*_1_ provided the best accuracy, AUC, and leave-one-out analysis AUC for all pediatric ages. The classification results indicate that IF can serve as a simple and sensitive tool for indicating risk of low LVEF. The proposed method can be done quickly, non-invasively, and easily, allowing for its use in multiple settings by both medical professionals and family members. This makes the IF method and Vivio device a potentially useful screening tool and valuable accessory to standard cardiac testing modalities. In the future, more diagnostic and prognostic information can be obtained from the IF method and smartphone-based devices such as Vivio as the hybrid IF-ML method is refined in pediatric patients by developing models for accurate LVEF quantification across all ages. This approach could be immensely useful for home monitoring or in resource limited medical settings where pediatric cardiac care is not readily available. This same methodology could also be adapted to calculate LVEF continuously from standard arterial blood pressure monitoring for critically-ill children in the hospital setting.

## Figures and Tables

**Table 1.** Demographics of patient cohort.

	Total (*n* = 50)	Normal LVEF (*n* = 34)	Abnormal LVEF (*n* = 16)
Sex, *N*(%)			
Male	22 (44%)	18 (53%)	10 (63%)
Female	28 (56%)	16 (47%)	6 (37%)
Race/Ethnicity, *N*(%)			
Asian/Pacific Islander	3 (6%)	3 (9%)	0 (0%)
Black/African American	3 (6%)	2 (6%)	1 (6%)
Hispanic	29 (58%)	17 (50%)	12 (75%)
Non-Hispanic White	12 (24%)	10 (29%)	2 (13%)
Other	3 (6%)	2 (6%)	1 (6%)
Age at examination, years (median, IQR)	9·9 (5·1–14·5)	9·2 (3·4–13·5)	12 (6·2–16·9)
BMI at examination, kg/m^2^ (median, IQR)	18 (15·3–24·1)	17·2 (15·5–23·3)	19·3 (14·9–26·7)
Diagnosis, *N*(%)			
Normal	20 (40%)	20 (59%)	0 (0%)
Status post cardiac transplant	7 (14%)	6 (18%)	1 (6%)
Status post chemotherapy	9 (18%)	8 (23%)	1 (6%)
Dilated cardiomyopathy	14 (28%)	0 (0%)	14 (88%)
Comorbidities, *N*(%)			
Moderate-severe mitral valve disease	2 (4%)	0 (0%)	2 (13%)
Diabetes	1 (2%)	0 (0%)	1 (6%)
Obesity	11 (22%)	6 (18%)	5 (31%)
LVEF (median, IQR)	59·1%(48–64·5%)	62·5%(58·9%−67%)	40·7%(21%−48%)
Receiving heart failure medications, *N*(%)	18 (36%)	6 (18%)	12 (75%)

**Table 2.** The classification performance of IF-based decision trees for low LVEF.

Evaluation methods	Predictors	Sensitivity	Specificity	Accuracy	AUC
Full sample analysis	*ω*_1_ *, age*	71%	94%	88%	0·87
	*ω*_1_, *age*, *φ*_1_	86%	92%	90%	0·91
	*ω*_1_, *ω****i***_1_	100%	89%	92%	0·95
Leave-one-out analysis	*ω*_1_ *, age*	64%	89%	82%	0·74
	*ω*_1_, *age*, *φ*_1_	64%	86%	80%	0·66
	*ω*_1_, *ω****i***_1_	93%	86%	88%	0·86

## Data Availability

All data that support the findings of this study are included within the article (and any supplementary information files). Data will be available from 2 February 2023.
